# Arabic speaking women’s experience of postpartum contraceptive counselling in Sweden

**DOI:** 10.1186/s12978-025-02074-2

**Published:** 2025-07-12

**Authors:** Sofia Berglundh, Khadija Abunnaja, Sibylle Herzig van Wees, Elin C. Larsson, Helena Kilander

**Affiliations:** 1 Department of Global Public Health, Karolinska Institutet, Stockholm, Sweden; 2 Department of Women’s and Children’s Health, Karolinska Institutet, Stockholm, Sweden; 3 Jönköping Academy for Improvement of Health and Welfare, School of Health and Welfare, Jönköping University, Jönköping, Sweden

**Keywords:** Family planning, Migrant, Contraception, Birth spacing, Contraceptive counselling, Shared decision-making, Midwife, Postpartum

## Abstract

**Background:**

Immigrant women living in Europe report lower use of contraceptives compared to native born women. The postpartum period is a key opportunity to provide high quality contraceptive counselling to support birth spacing, but little is known on how the counselling could be adapted to meet the needs and preferences of immigrant women. Approximately a third of all women giving birth in Sweden have an immigrant background, whereof Arabic speaking women constitutes one of the largest groups. Hence, the aim of this study was to explore Arabic speaking women’s perspectives of contraceptive counselling postpartum.

**Method:**

Five focus group discussions (FGDs) were conducted with 23 Arabic speaking women. The FGDs were conducted in Arabic and translated to English. Data was analysed using reflexive thematic analysis.

**Results:**

Four main themes were created: 1) *Adapting to new circumstances influence reproductive intentions:*raising children in a new setting was described as a double burden and birth spacing was seen as essential for the family’s wellbeing. 2) *Reproductive decision-making - the women's choice but partner’s* *support is important:* inviting the partner to the contraceptive counselling was thought to enhance both his knowledge of contraceptives and his understanding of the woman’s entire life situation postpartum. 3) *Conflicting information about contraceptives creates hesitancy:* navigating opposing information on contraceptives from the woman’s home country and midwives in Sweden was confusing and fears of negative side effects from contraceptives were deep-rooted. 4) *Trust and mistrust in antenatal and postpartum contraceptive services: *trust included experience of emotional support and an open-minded attitude from the midwife. Mistrust involved scarce support in handling side effects, limited decision support and a feeling of breached privacy.

**Conclusion:**

To provide person-centred and equitable contraceptive counselling postpartum, health care services need to shift attention from individual barriers to how the counselling can be improved. Key elements include integrating the concept of birth spacing in the postpartum contraceptive counselling, ensuring accessible follow-up services and to provide comprehensive information in the native language to support informed choices. An open-minded engagement with patients is also central to provide contraceptive counselling that is inclusive for all women.

## Abstract (plain language summary)

The period after childbirth is a key opportunity to support women’s choices on contraception and family planning. Women with an immigrant background are less likely to use contraception, yet little is known about how contraceptive counselling can be adapted to meet their specific needs.

In this study we explored Arabic speaking women's experiences of, and preferences for, contraceptive counselling after childbirth in Sweden. Five focus group discussions were performed with 23 Arabic speaking women. Women described that adjusting to circumstances in a new home country changed their views on family planning. Raising children in a new setting was described as difficult and sufficient time between pregnancies was essential for the family’s wellbeing. It was the woman's choice to use contraceptives, but the partner’s support was important. Inviting the partner to the contraceptive counselling was thought to increase his knowledge of contraceptives and his understanding of the woman’s situation after giving birth.Handling conflicting information on contraceptives from the woman’s home country and midwives in Sweden was confusing and fears of side effects from hormonal contraceptives were deep-rooted. Many women described trustful experiences of contraceptive counselling. But limited support in handling side effects, too little guidance in choosing type of contraceptive method and a feeling of privacy breach were examples of mistrust. Adjustments to contraceptive counselling is needed to achieve individualised contraceptive counselling after childbirth. This includes inviting to dialogues about birth spacing (time between pregnancies), ensuring accessible follow-up services and providing detailed information in the native language.

## Background

Sexual and reproductive health and rights (SRHR) are fundamental for women’s health and to promote gender equality [1]. This includes the freedom to decide if, when, and with whom to have children. Access to contraceptive counselling postpartum is crucial to prevent unintended pregnancies [1], and research shows that contraceptive choice in the postpartum period is associated with a lower risk of abortion 12–24 months after childbirth [2].

However, contraceptive services seem to fail in assisting immigrant women that have resettled to high income countries to access contraceptive methods of their choice [3–5]. Research shows that immigrant women report lower use of contraceptives [3, 4] and have a higher incidence of induced abortions [6–8] compared to women in the majority population in Europe and North America. Moreover, contraceptives are less frequently prescribed to migrants and refugees compared to native-born residents according to a Dutch study [9].

The evidence on how contraceptive counselling could be adapted to ensure inclusivity for immigrant women is scarce [10]. *Postpartum* contraception is an even more complex field, where aspects like breastfeeding, birth spacing, timing of contraceptive counselling (e.g. antenatally or postnatally) and medical conditions post births must be considered [11]. A recent scoping review on patients’ preferences for perinatal contraceptive counselling highlights the need of individualised care, with flexibility in both the timing and the content of counselling [12]. Others conclude that detailed information and support for the woman’s reproductive autonomy are key factors [13]. Both reviews point out the lack of research focusing on minority women’s preferences for contraceptive services postpartum [12, 13].

In general, individual barriers to contraceptive use among immigrant women have on the other hand been extensively explored [14–16]. Negative experiences of contraceptive counselling and misconceptions about hormones have been suggested as reasons for lower contraceptive use among immigrant women compared to native born in various contexts [14, 15]. Other obstacles to contraceptive use include insufficient knowledge about sexual and reproductive health (SRH) [14, 16] and communication and language barriers [14, 15]. The partner’s influence on contraceptive use is still debated. Studies show that patriarchal structures can hinder female reproductive autonomy [14, 17], meanwhile others conclude that involving men in the contraceptive counselling can facilitate contraceptive access and use [18], thus strengthening women’s SRH. Immigrant women’s views on partner involvement are less well known.

A few studies highlight the healthcare provider’s role in contraceptive uptake among immigrants [19, 20]. Dehlendorf et al., have shown a relationship between high quality interpersonal contraceptive counselling and continuation of chosen contraceptive method [20]. Similarly, a Swedish study report that trust between the midwife and patient is paramount in achieving fruitful contraceptive counselling [19]. Conversely, structural racism in health care exists [21] and experience of disrespectful care and discrimination constitutes barriers for SRHR, including Islamophobia and presumptions about Muslim women’s lack of autonomy [15].

Over the past 20 years, migration to Sweden has increased [22] and approximately a third of all women giving birth in Sweden have an immigrant background [23]. In 2021, more than 3.6% of the population originated from Arabic speaking countries. Syria and Iraq, represents some of the most common birthplaces of foreign-born residents in Sweden, (1.9 and 1.4% of the population respectively) [24]. Considering the recent pattern of immigration to Sweden, Arabic speaking women are an important, yet diverse group to consider in reproductive health research.

There is a current public health focus on strengthening women’s health in Sweden, particularly regarding equity and SRHR [25]. However, to accomplish person-centred contraceptive services that are inclusive for *all* women living in Sweden and other similar settings, the end-users’ perspectives on reproductive agency, choices, and needs must be in focus. This study was conducted within the IMPROVE-it project, which aims to improve postpartum contraceptive services for, and with, immigrant women in Sweden [26]. This part of the IMPROVE-it project was focusing on Arabic speaking women, and findings from this study were used in workshops with the target group to develop and refine a quality improvement intervention, further described in the IMPROVE-it study protocol [26].

Hence, the aim of this study was to explore Arabic speaking women’s perspectives of contraceptive counselling and their experiences of *postpartum* contraceptive counselling, as well as the partner’s involvement in contraceptive counselling.

The following research questions where explored:

- *What are Arabic speaking women’s views on contraceptive counselling in general, antenatally and postpartum?*
- *What are the views on partner involvement in contraceptive counselling?*
- *How can postpartum contraceptive counselling best be improved?*

## Method

### Design

This was an exploratory qualitative study, using focus group discussions (FGDs). FGDs involve bringing together a small, homogenous group of individuals to discuss a particular topic in a facilitated group setting, and provides an opportunity to gather information on attitudes and experiences related to a specific issue [27].

This study has its roots in social constructivism. From a social constructivism perspective multiple realities exist; knowledge is socially constructed by human activity and knowledge can change the reality [28]. We assume that possessing knowledge on evidence-based methods for contraception is not enough to provide good family planning; the interaction between patient and health care staff is also of importance as well as acquiring knowledge regarding women’s preconceptions, experiences and beliefs that might influence her choice of contraceptive method.

### Study setting

In Sweden all women are offered routine postpartum visits at a maternity health clinic 4–16 weeks after giving birth [29]. The number of postpartum visits (one or two) varies based on the region and the individual needs of the woman. The visit is usually with the midwife who managed the antenatal care, it is free of charge and contraceptive counselling should be offered during this visit. In this study, contraceptive services were defined as counselling, (carried out antenatally, postpartum or both), prescription and/or administration of contraceptive methods.

### Sampling procedure

Purposive sampling and snowball sampling were used to recruit participants [30]. The sample was selected based on the following inclusion criteria; women who are immigrants, Arabic speaking, >=18 years old and have experienced childbirth in Sweden. Participants were recruited via several channels, including open pre-schools (public playgroups), Swedish language schools, mosques, social media groups and family health centres. The two research assistants (one of them co-author KA) were instrumental in identifying potential participants and organizing the FGDs at a convenient time and location for the participants. In addition, when participants were confirmed for participation in the FGDs, they helped identify other potential participants to the study. Five FGDs were conducted, of which four were face-to-face and one online. Each FGD had a varying number of participants, ranging from three to seven individuals. A total of 23 women participated in the study.

### Data collection

The data was collected in Stockholm, Sweden between April, and October 2022. The FGDs were conducted in Arabic, the native language of the participants and the moderator. A FGD guide was used, which was pilot tested and only minor adjustments were made. We reflected on the richness of the data after familiarising ourselves with the five transcripts and deemed the data to then be sufficiently rich. The data collected in the pilot was kept in the analysis since it was considered as rich data.

In two of the FGDs an Arabic speaking observer participated, and in one FGD a non-Arabic speaking observer participated. In the other two, one of the first authors (KA) facilitated the FGDs on her own (one online and one live) due to practical reasons. The moderator established a good connection with the participants, took field notes during those sessions and any reflections were discussed within the team after the session. However, we cannot exclude that some subtle interactions were missed when an observer wasn’t present. Both online and live FGDs worked well, with natural group interactions, open sharing and without technical issues. The FGDs were held in private spaces (closed rooms) and the moderator explained the confidentiality process of the data (recordings) as well as the importance of respecting different opinions and views in the discussions and that there were no right or wrong answers.

Observation notes were collected during all FGDs by the observer or moderator. The duration of the FGDs ranged from 66 to 96 min, with a median of 70 min. The participants gave their written informed consent for participation in the study. Background data on parity, sociodemographic factors and previous contraceptive use was also collected, using a short participant information sheet. The characteristics of the women are described in Table 1.

**Table 1:** The participant’s demographic background, obstetric history and previous contraceptive use before and after last childbirth

Characteristics	Count or median*
Number of participants	23
Number of FGDs	5 (1 online and 4 in person)
Number of years living in Sweden	7 (1–18)*
Age (yrs)	32 (24–44)*
Highest education attained	
* Below upper secondary school*	3
* Completed upper secondary school*	11
* Completed University*	9
Employment	
* Studying*	12
* Working*	6
* Parental leave*	2
* Other*	3
Home country	
* Syria*	11
* Egypt*	3
* Iraq*	4
* Jordan*	2
* Libya*	1
* Morocco*	1
* Palestine*	1
Marital Status	All are married.
Number of pregnancies	3 (1–9)*
Number of children	3 (1–6)*
Number of abortions	0 (0–3)*
Previous use of contraceptives *before* the last childbirth	
* Yes*	17
* No*	5
* Do not know*	1
Type of contraceptive methods used *before* last childbirth	
* IUD*	8
* Pills*	9
* Skin patch*	3
* Condom*	4
* Safe period*	3
* Interrupted intercourse*	1
Recent use of contraceptive *after* the latest childbirth	
* Yes*	17
* No*	6
Type of contraceptive methods used *after* last childbirth	
* IUD*	7
* Pills*	3
* Skin patch*	2
* Condom*	3
* Safe period*	4
* Ovariectomy*	1

*Median (range)

### Data management

The FGDs were audio-recorded, transcribed verbatim in Arabic, and translated to English by the moderator of the FGDs (KA). A subset of the translations was back translated and deemed to be of good quality. Transcripts were anonymised and stored together with the audio recordings in a two-way authentication encrypted drive, only accessible by members of the research team.

### Data analysis

Reflexive thematic analysis, guided by Braun and Clarke [31] was employed to analyse the data, using an inductive approach. Thematic analysis is a flexible and commonly used method that allows for the identification, analysis, and reporting of patterns (themes), providing a rich and detailed interpretation of the information [31]. All the transcripts were first double coded independently by the first authors (KA, SB) to get a broad, nuanced and detailed understanding of the data. Codes were then repeatedly discussed and refined by first authors with guidance from SHvW and HK, creating a joint coding tree. A collaborative process was then employed to explore patterns and meanings in the data, combining codes into subthemes and themes. The themes were repeatedly discussed, challenged and revised to make sure it captured the complexity of the data. Themes were agreed on by all authors (KA, SB, HK, SHvW, ECL). The participants’ background information, as presented in Table 1, was considered during the analysis to better comprehend their context and experiences. NVivo software was used to support the coding process. A member check was performed during workshops as part of the IMPROVE-it project described in Study Setting. In the workshops, findings from the FGDs were shared with participants to ensure mutual understanding of the data.

### Researcher characteristics and reflexivity

Throughout the research process, reflexivity plays a vital role; where researchers reflect on their preunderstandings, values, and experiences and how these may influence the research process and interpretation of the data [32]. The members of the research team have diverse backgrounds (researchers in SRHR, midwife, medical doctor, associate professor in global health/SRHR, with extensive experience of migrant health and/or experience of qualitative research methods, cultural knowledge, and relevant languages skills). The first author’s (KA) background, as an Arabic speaking woman who recently moved to Sweden with her family and with a Master’s in Global Health, helped in her understanding of the participants’ experiences. This shared background facilitated the recruitment and enabled honest discussions as participants may have felt more comfortable sharing their experiences with someone who understands their cultural context and has faced similar challenges. However, the researcher’s background also had potential implications for the data interpretation. To address this, two researchers independently coded the data. The other first author (SB) has a background as a medical doctor with experience of working in gynaecology. This could be an asset by having knowledge of the topic and experience of providing contraceptive counselling to immigrant women. However, it could also introduce a power imbalance and preconceived ideas. By not moderating the FGDs, SB could stay distant from her role as healthcare professional, minimising the risk of introducing assumptions in the discussion. The other co-authors added their perspective during the later stages of analysis, which further strengthened the reflexive process in the data interpretation and analysis.

### Ethical considerations

The Swedish Ethical Review Authority has approved the study (Dnr 2020–05710). The participants received written and oral information in Arabic, both during recruitment and at the time of the FGD. They were informed that their answers were anonymous, that they could withdraw at any time, that participation was voluntarily, and the purpose of the study. Informed consent was obtained for each participant at the time of the FGD, including consent for audio recordings.

There are some considerations that warrant specific attention in this study. Immigrant women can be in vulnerable situations for many reasons, including language barriers and the need to navigate a new health system [33]. There is also a risk of stigmatising by attributing immigrant groups collective views on SRHR and contraception. However, the focus of this study was on preferences for contraceptive counselling postpartum, not personal contraceptive behaviour and this was explained during the recruitment process. The purpose of the study was to increase the knowledge in this area to provide more equitable sexual and reproductive care for the target group, thus the findings are expected to benefit the participants on a group level.

## Results

**Fig. 1:**
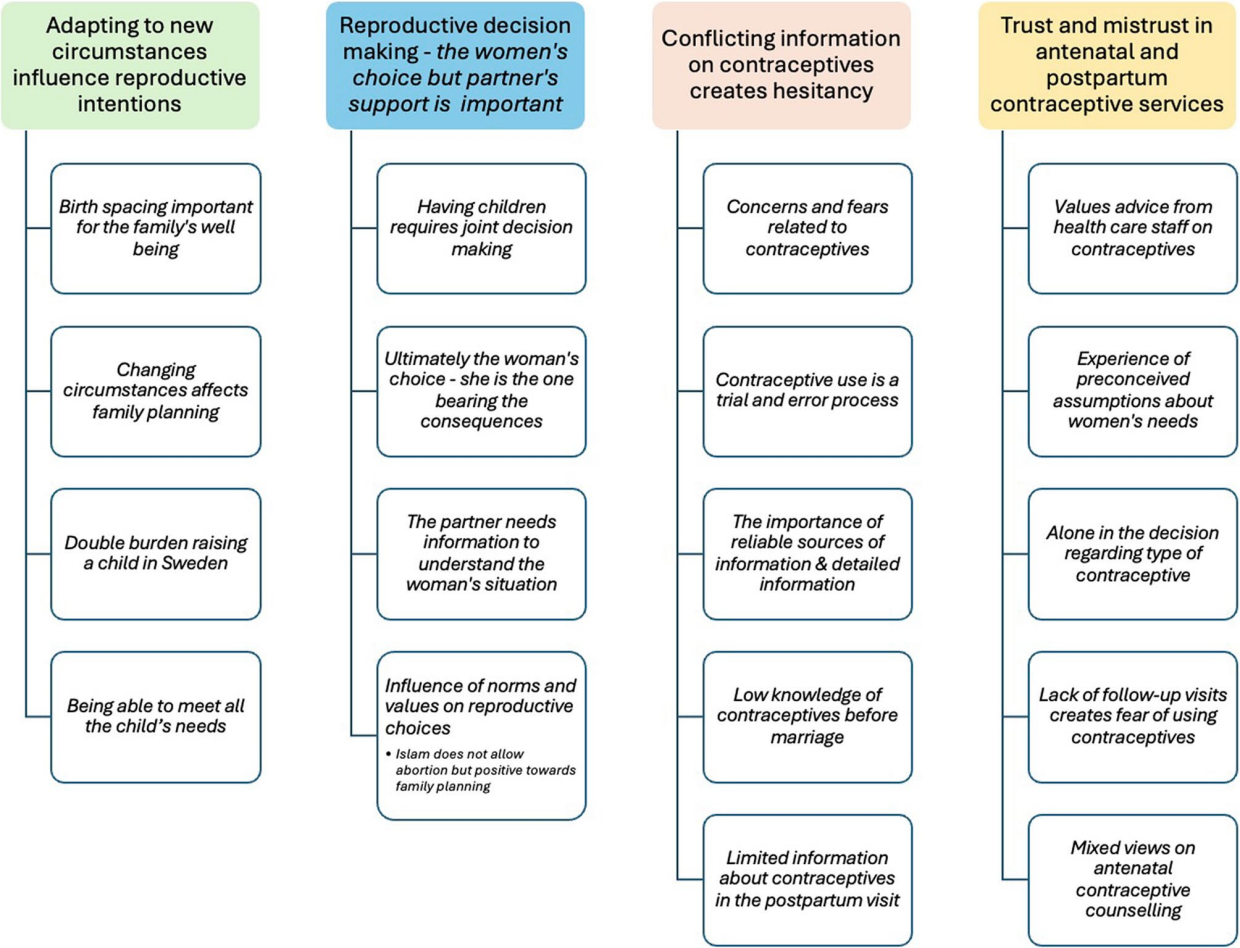
Main themes and subthemes that emerged from the focus group discussions

Four main themes were created; 1*) Adapting to new circumstances influence reproductive intentions;* (2) *Reproductive decision-making - the women’s choice but partner’s support is important;* (3) *Conflicting information on contraceptives creates hesitancy;* and (4) *Trust and mistrust in antenatal and postpartum contraceptive services.* Each theme includes four to five sub-themes, see Fig. 1. Below, the themes are described and illustrated with selected quotes. In brackets the number of the FGD, and the individual are presented as follows: (FGD1, P2). The participants will be referred to as women from here on.

### Adapting to new circumstances influence reproductive intentions

This theme highlights how women’s changing life circumstances, following migration to Sweden, changed their reproductive intentions. Women were hesitant to having children in times of uncertainty and various aspects were considered in the reproductive decision-making.

Raising a child in Sweden, compared to country of birth, was expressed as a double burden, with added responsibilities for the woman besides childcare. Aspects of the integration process, like learning a new language, working, and studying were identified as putting pressure on the family and affecting the desired family size. Being more isolated in raising children in Sweden was also brought up as a challenge, referring to not having the extended family close by for support.

*Yes*,* it is very true*,* especially as we are in a new country*,* we want to learn the language and expand our knowledge. In other words*,* I am a Syrian university graduate from the Faculty of Law*,* when I moved to Sweden*,* I had to start from zero*,* so I needed to improve myself because if I didn’t*,* I’d be mentally exhausted. So*,* all these factors influenced my decision to become pregnant.* (FGD1, P1)

The main priority for the women was being able to provide secure conditions for the children they already had, including good housing and financial stability, as well as being able to devote sufficient time and emotional support to each child.

*I came to Sweden a year and four months ago*,* and my house is small. I cannot have another child at the present time. I must work so that we can get a loan and buy a house.* (FGD1, P3)

Many of the women described that the responsibility for childcare fell on them, impacting their life choices as well as their mental health. As a way of managing the increased stress from raising children in a new setting, birth spacing was described as essential for both the woman’s and the entire family’s wellbeing. On top of a stressful life situation, the women also highlighted the need to let the body rest between pregnancies and expressed a wish that midwives would invite to dialogues about the various benefits of birth spacing.

*I think the midwifes can help*,* they can explain to women how the situation is with one child with two or three children./……/Meaning they should explain that the more children you have*,* the more it will drain your ability*,* and your interest in the rest of the children will decrease./………/I see that she needs to provide more information about having children and its impact*,* I mean. (*FGD1, P1)

However, a few women also described that being alone in new context could be a reason for wanting a larger family, since children can provide a sense of belonging. The concept of planning your pregnancies was seen as fluctuating, e.g. deciding your family size ahead is not always feasible, since you do not know how your circumstances might change.

In conclusion, living in the new Swedish context made women think more about contraception. However, this was not only the women’s decision as presented in the next theme.

### Reproductive decision-making - the women’s choice but partner’s support is important

Most women shared the view that contraceptive use is a joint matter concerning both the woman and her partner, and the number of children and contraception were regularly discussed with the partner. However, despite considering the partner to have a significant role in the reproductive decision-making, women also expressed that using contraceptives and or becoming pregnant should be the woman’s choice in the end. The women rationalized that the partner does not need to endure side effects, and that it is the woman that bears the consequences and risks with pregnancy and childbirth, thus it should be her final choice.

*As for contraception*,* the first and last decision is for a woman*,* because she is the one who gets pregnant*,* gets tired and gives birth*,* and sometimes contraceptive methods are not suitable for her body*,* and the woman becomes like a field of experiment*,* unfortunately.* (FGD4, P1)

Women in this study were in general positive to involving the partner in the contraceptive counselling. They stated that including the partner was important to increase men’s, according to the women, often limited knowledge on contraceptives, hormones, and reproductive health. Another important aspect of involving the partner was to expand his understanding of the woman’s life situation. The women expressed a great need for the partner to better recognize how pregnancies, childbirth, and contraceptives (e.g. hormones) affect the woman’s body and wellbeing. Inviting the partner to the contraceptive counselling and receiving information from the midwife was thought to enhance the partner’s support and help emphasize shared responsibility of contraceptive use.

*I mean*,* I think*,* in order for him to have a background in contraceptives*,* so if we plan not to have children*,* it is good for him to hear from the midwife about contraceptives and their effect on my body. Planning for childbearing is not only the responsibility of the woman*,* but the responsibility of the man as well and the decision is shared between them*,* whether to become pregnant or stop childbearing.* (FGD5, P3)

However, a few women preferred not to include the partner in the contraceptive counselling session, due to privacy reasons or that the partner could be embarrassed by the sensitive topic. Furthermore, religion can influence reproductive choices for some. A few women explained for example that Islam does not allow abortions but supports birth spacing.

*It is not a mistake to have many children*,* but our Islamic religion also advised us to distance between pregnancies*,* as the woman should take a break between them*,* at least three years*. (FGD3, P3)

In summary, men’s involvement in postpartum contraceptive counselling was seen by many as an opportunity for the couple to access information needed to support their reproductive choices. The importance of detailed information in the contraceptive counselling is further described in the next theme.

### Conflicting information about contraceptives creates hesitancy

Conflicting information from friends, family, and healthcare providers in the woman’s home country, compared to information from midwives in Sweden was perceived as confusing and created a feeling of not knowing what information to trust. The contraceptive knowledge prior to moving to Sweden was to a large extent from family and friends, and pre-marital contraceptive counselling was described as uncommon.

A wish for more detailed information about various contraceptive methods during counselling was expressed, and the women explained that insufficient information could lead to misunderstandings and incorrect use of contraceptives. There was a request for more comprehensive information on side effects, health benefits, and risks. Women preferred to receive information from reliable sources, written information was highly appreciated and some women also found information from websites like the Swedish national health advice homepage helpful.

*…regarding the use of the new type of contraceptive pill*,* the midwife gave me many papers that talk about this contraceptive in details. This is a very good thing*,* because this written information is from a reliable source*,* so I read it and I am confident*,* not like what I read from the internet*,* for example.* (FGD5, P2)

However, some women believed that even detailed information was not enough; the woman need to try the contraceptive herself to see whether it fits the nature of her body. They acknowledged that different methods work well for some women but not for others, and described that it was often difficult to find a suitable contraceptive method. This trial-and-error process (trying many different contraceptive methods) could be frustrating according to the participating women.

… *[I] also agree that according to the nature of the body*,* for example*,* the pills so far are very good for me*,* while neither the IUD (intrauterine device) nor the skin implant suited me. My body did not accept anything from outside*,* such as the IUD*,* so the pills suited me very well*,* and on the contrary*,* they did not cause me nervousness or weight gain.* (FGD5, P4)

Fears about hormones were commonly described, and women expressed concerns about side effects such as cancer, amenorrhea, infertility, and anxiety. Hormone use was seen as scary and harmful for the body. Previous negative experiences of contraceptives often influenced the women’s contraceptive choices, as well as experiences and advice from friends and relatives.

*Frankly*,* the pills have side effects*,* obesity*,* headaches*,* as well as the IUD*,* they say that it causes cancer*,* so I am very far from using any type of contraceptives*. (FGD 2, P3)

The fear of infertility was particularly strong, and some women had been advised by both family and health care providers in their home country not to use any contraceptives before giving birth to their first child.

*It can cause harm when used from the beginning of the marriage before pregnancy occurs*,* infertility may occur. From the experience of one of my friends in Jordan*,* she got married and wanted to use birth control pills*,* so she consulted*,* and they told her not to use them from the beginning*,* it would be better to have a child and then think about using it*. (FGD2, P3)

Some women expressed that the midwife has an important role in dispelling myths about contraceptives and to reassure about contraceptives and health concerns. One woman said it would be useful with educational lectures and even suggested mandatory appointments after childbirth to give information about contraceptives. Others said that “*It is necessary that the midwife perform her role at the fullest and give a lot of information to the women”.* (FGD4, P1).

*Frankly*,* sometimes these questions that we also ask ourselves*,* the fact that contraceptives sometimes cause infertility? Does it really cause cancer*,* as many people are talking about? I really feel that this information is incomplete*,* and the midwife needs to explain more about it* (FGD5, P1)

A lack of trust in the effectiveness of contraceptives was also discussed. Some women had either experienced themselves, or heard from others, that contraceptives sometimes fail, resulting in unplanned pregnancies. This was described for both modern contraceptives and natural methods. Thus, both fears for side effects and rumours were barriers for contraceptive use. However, some side effects were perceived as acceptable if the contraceptive method was efficient to prevent pregnancies. The women also discussed that they were left with no other choice than to accept side effects, despite experiencing a negative impact on their body or mental health.

*Frankly*,* from my experience*,* it [contraception] is absolutely not good and harmful. I tried contraceptives and suffered greatly./……./I was always in appointments and hospitals*,* and you know here in Sweden everything is slow. From my point of view contraceptives are bad*,* but I have to use it as well*,* as all women have to use it to avoid pregnancy*. (FGD5, P4).

Natural contraceptive methods were the first choice for many women, especially for women with previous negative experiences of hormonal contraceptive methods. Several women were satisfied with the use of natural methods and stated that the method had worked well for them for years. Despite being a common choice, information on natural methods was absent from the contraceptive counselling and was specifically asked for to be included.

*Educate us on the natural method of contraception and the method of counting*,* we know it and we can read about it from the internet*,* but she [the midwife] can give us more reliable information for sure.* (FGD4, P2)

Despite wanting to use contraceptives, fear of hormones was common, and women asked for more comprehensive information on side effects and natural methods. Another aspect discussed was the importance of establishing trust - both in the relationship with the midwife and at an organisational level– and this will be presented next.

### Trust and mistrust in antenatal and postpartum contraceptive services

While many women reported trustworthy contraceptive counselling by their midwives, there were also some experiences of mistrust in the antenatal and postpartum period. Trust included an empathetic counselling experience. Mistrust involved limited support in handling concerns about side effects of contraceptives, limited decision support, too little focus on the woman’s health postpartum and a feeling of breached privacy.

To receive contraceptive counselling antenatally was met with mixed feelings. Many were positive since it allowed some time for the woman to think about it, to prepare and plan. It was also seen as a useful tool for supporting birth spacing. Others thought antenatal counselling was overwhelming and one woman described it as; “*I honestly feel that the issue is a bit difficult during pregnancy*,* I think of childbirth and its troubles.”* (FGD4, P1).

Some women felt that midwives had preconceived ideas about their needs as immigrant women, especially in the sensitive time of postpartum, when the women had other priorities and expectations. Women felt that other things were more important, for example advice on breastfeeding. Another example was midwives asking questions about their relationship and domestic abuse rather than focusing on the woman’s physical health after giving birth. Some women saw personal questions about the (male) partner as interfering with their private life, affecting their trust in the midwife, which in turn could hinder the dialogue in general.

*Frankly*,* I do not like questions of the investigative type. I mean*,* for example*,* is your husband violent with you? Is there something going on with you that you would not like to talk about in front of your husband? These questions are very annoying and unacceptable to me*. (FGD4, P3)

On the other hand, some women stated the opposite, saying that it was the midwife's obligation to ask about these matters and appreciated that it was brought to attention. Many women also described experiences of the midwife being understanding and mindful of cultural differences.

*Honestly from my experience*,* I feel that she is very understanding and understands that I am a Muslim woman*,* and I have my own customs and traditions. For example*,* when she asks me*,* do you drink alcohol*,* she tells me that I know that you are Muslim and you wear the hijab*,* but I just have to ask*,* I mean*,* I feel that the midwives are very understanding and educated in this regard.* (FGD5, P4)

Emotional support from the midwife was mentioned as important in the antenatal and postpartum period, both in general but also in relation to contraceptives. Some women expressed that their mental health was very important to them, and that concerns about contraceptive's effect on mental health should be embraced in the contraceptive counselling.

*Ok*,* it is not wrong to ask about my psychological condition*,* for example*,* and whether I am nervous by nature or not*,* because for example*,* it is possible for contraceptive pills to increase nervousness and the situation to get worse.* (FGD5, P1)

The women agreed that the midwife does not interfere in the decision regarding type of contraceptive method, explaining that the midwife would provide information about each type of contraceptive, but would not advise the woman to select a specific type. Experience of patient-driven decision-making could sometimes create a feeling of being lonely when choosing a contraceptive method. One woman described that she received information about all available options and their effect, but that the midwife did not “*offer any other help*”, it was up to the woman to decide “*according to the nature of her body”* (FGD2, P1).

*I think that the midwife does not interfere in these matters at all. For example*,* I asked her (what do you think about the skin implant? And what is your advice?)*,* she explained to me how it works*,* and she said that she had nothing to do with my decision*,* and she could not advise me on a specific thing. She was so impartial*,* it means she has nothing to do with the matter.* (FGD1, P1).

Women that had received advice on certain methods from the midwife perceived it as helpful, explaining that the midwife’s opinion was important in making their choice, without experiencing feelings of being pressured: *“Just advice*,* I mean she doesn’t force you”.* (FGD3, P1)

Some other examples of mistrust in contraceptive counselling were also discussed among the women. A difficulty to book a health care appointment in Sweden and long waiting times were considered barriers to contraceptive access and use. This created a hesitancy of using contraceptives, since the women were not confident that they would receive timely help in case of side effects. Many also expressed concerns about the lack of routine check-ups of intra uterine devices (IUDs) to confirm correct position, making them hesitant to insert an IUD in the first place.

*I am not talking about pregnancy*,* but sometimes I feel that I have pain from the IUD*,* and I want to meet her (the midwife)*,* I call but I do not get an appointment until after a long time. (*FGD3, P2)

## Discussion

The aim of this study was to explore Arabic speaking immigrant women’s experiences of, and preferences for contraceptive counselling postpartum. Our results show that women in general were positive to using contraceptives, mainly to support birth spacing, but not at the expense of their physical or mental wellbeing. Generally, women had knowledge of different contraceptive methods but navigating conflicting information on contraceptives from home country and midwives in Sweden contributed to hesitancy to use certain methods, in particular hormonal ones. Involving men in postpartum contraceptive counselling was largely seen as an opportunity for the couple to access trustworthy information needed to support their reproductive choices. Women also expressed a need for more support in the decision-making process of choosing a suitable contraceptive, as well as more assistance in managing side effects.

Our results differ somewhat from previous literature, which have focused mainly on the individual barriers in the utilisation of contraceptive services amongst immigrant women, such as low reproductive knowledge, negative attitudes, low autonomy or cultural and religious beliefs [14, 16]. Our results show that many Arabic speaking immigrant women have positive attitudes to contraceptives and that cultural and religious beliefs can have a rather small influence on their contraceptive choices. This study rather emphasises the importance of providing contraceptive services as a continuum of care, from receiving information, through decision-making, to follow-up support, as also described in the Person-Centered Contraceptive Care framework by Holt et al. [34]. Thus, although there were many positive experiences of postpartum contraceptive counselling, there is room for improvement to support immigrant women’s reproductive needs. This also includes a non-biased approach in the counselling.

The women in our study described that their reproductive intentions changed in response to changing life circumstances after migration. Birth spacing was described as motives for contraceptive use to ensure good living conditions, wellbeing for the family, and to allow time for the integration process in a new country. The routine postpartum visit in Sweden is an opportunity to reach many women with contraceptive counselling. However, the postpartum period could also be a sensitive time, and person-centeredness is therefore particularly important [12]. The quality of the counselling needs to be high, and the woman needs to be at the centre of the counselling. By shifting focus from *preventing* pregnancies, to strengthening women’s reproductive choices, the counselling could be more inclusive. A trustful counselling could further be achieved by asking open ended questions, focusing on concepts like *birth spacing* and *reproductive life plan* (i.e. “*Do you wish to have more children*,* and if yes*,* when?*) rather than solely focusing on contraceptive use. Acceptability of child spacing as a concept has been reported previously [35] and could facilitate dialogues between the woman and midwife.

A challenge described in our study was handling conflicting information about contraceptives. Advice from midwives in Sweden was seen as trustworthy, yet rumours were common. This considerable fear of hormones has been described previously and is not by any means unique for Arabic speaking immigrants [36]. Providing verbal information at the postpartum visit does not seem to be sufficient to make women feel confident using hormonal methods. This could be counteracted by providing written information in the native language, or by translated video material, including positive health aspects of contraceptives. These approaches have been shown to be facilitators in providing reproductive health care for migrant women [37]. However, the education level was high in this study, which could explain the wish for written information, and might not be considered helpful for women with lower education level.

Women also asked for more education on natural methods - a method often dismissed by health care providers (probably due to its low effectiveness [38]). However, avoiding discussing this method is a missed opportunity to provide trustworthy information about this, and other contraceptive methods, while respecting the woman’s preferences and needs, which is fundamental in person-centred care [34].

The contraception counselling in Sweden was described as often being neutral, i.e. the midwife does not interfere with the choice of contraceptive method. Protecting the woman’s reproductive autonomy is crucial and perceived pressure to choose a certain method is related to discontinuation and low patient satisfaction [39]. However, some women experienced this neutral approach to counselling as a lack of support in the decision-making process and expressed that the midwife’s expertise was both valued and explicitly requested. We speculate that moving from a context where provider driven decision-making is common, to a purely patient driven decision-making system could sometimes be overwhelming. Furthermore, the absence of advice on method selection may sometimes be perceived as a lack of competence on the midwife’s part, suggesting uncertainty about what is best for the patient, as described by Kolak et al. [40]. Shared decision-making (SDM) could have some advantages [41], with the woman feeling more supported in choosing a method that suits her needs, rather than receiving detailed information on each method. Guidance based on the woman’s preferences is an example of SDM [41] and has been reported to be perceived as helpful by immigrant women [19]. Research also shows that women who have received SDM counselling are more likely to be satisfied with their chosen contraceptive method [42]. However, the term SDM is somewhat problematic as it implies that the provider should to take part in making the decision. An alternative approach is informed choice, in which the midwife supports individuals in making voluntary, well-informed decisions about contraceptive methods based on accurate, unbiased, and comprehensive information [43]. The key distinction between SDM and informed choice lies in the level of interaction during the decision-making process [44]. The most suitable approach may vary depending on the patient’s needs and preferences, but the final decision should be the woman’s.

The need of ensuring follow-up support as part of contraceptive services was evident in this study. The perceived difficulty to book an appointment for health check-ups was described as a barrier for contraceptive use. Fear of not receiving timely help to manage side effects or for method switching can refrain women from starting a contraceptive method in the first place, especially provider-controlled methods [45]. Thus, women’s concerns for side effects must be taken seriously and easier access to LARC removal need to be ensured to support women’s reproductive autonomy. Women in our study also expressed a wish for ultrasound check-ups of IUDs, which is a common approach in some of their home countries. A lack of similar follow-up in Sweden created hesitancy to use this method. It is thus of importance to give thorough information on *why* this procedure is not considered necessary to avoid mistrust in contraceptive services.

The importance of a trusting and non-judgmental relationship with midwives found in this study aligns with qualitative research from Australia and Sweden [19, 46, 47]. In Sweden, midwives are often the first contact for migrants within the healthcare system, and a positive first encounter can help establish long-term trust in health services [48]. However, healthcare providers often report limited knowledge of immigrant women’s specific health needs, which poses challenges in contraceptive counselling and other SRH services [49]. Therefore, enhancing training for health professionals is a crucial step in improving the quality and accessibility of reproductive healthcare for immigrant women [50]. Minimising preconceived ideas about the patient is crucial to avoid mistrust in health services. Religious beliefs have, for example, traditionally been labelled as barriers to contraceptive use [14], however the reality is more complex. In this study, we found that religious beliefs did not significantly hinder the use of contraceptives, in fact, it was rarely mentioned to influence reproductive choices. A few women mentioned that Islam supports birth spacing. Thus, religion could act as an enabler for contraceptive use for some, but not all Muslim women, and this has been described previously [51]. Our findings challenge common stereotypes about Muslim women and emphasize the need for healthcare approaches that are informed by voices from the target group rather than assumptions about women’s beliefs and needs. It is thus time to focus more on health system changes, including bias training for health care providers to achieve person-centred contraceptive services [34]. This also holds true for partner involvement. Previous literature has described hesitancy among health care staff to involve the partner due to fear of compromising the woman’s autonomy [15]. However, in this study most of the women were positive to inviting the partner to the contraceptive counselling, as family planning was seen as an issue that concerns the partner. The women also expressed a need to increase men’s knowledge on the contraceptives’ effect on women’s health, as well as their overall understanding of women’s situation postpartum. Recent research from Sweden exploring immigrant women’s and men’s perspectives on contraceptive counselling reveals similar findings, showing a positive attitude towards involving men in the process [19, 52]. Joint visits incorporating voluntary couple-based counselling could thus potentially serve as a valuable complement to individual contraceptive consultations. By inviting the partner, shared responsibility for contraceptive use could also be emphasized [15]. However, male involvement is not favourable for all women, hence asking the woman about her preferences and obtaining her consent before inviting the partner is always imperative to ensure reproductive autonomy.

Findings in this study do not only describe Arabic speaking women’s views on contraceptive counselling but have practical implications for how contraceptive services could be improved to facilitate person-centred care and contribute to increased equity in reproductive health care. Overall, contraceptive access programs are undergoing continuous development, from fertility control to a growing emphasis on human rights, informed choices, and equity as outlined in global [43, 53] and national [25] policies. Our findings align with these strategies, underscoring the need to adapt services to meet diverse perspectives and needs in reproductive care by incorporating bias training for health care providers, providing comprehensive information in multiple languages to enhance informed choice, expanding follow-up support, and shifting the focus toward reproductive choices throughout the lifespan rather than a single-session approach. Future research could benefit from gaining more insights on aspects like outreach and trust building in the community. This could include exploring acceptability for contraceptive group or couple counselling session and other outreach efforts.

### Methodologic considerations

Performing the FGDs in Arabic by a native speaker is major a strength of this study. This approach reduced the risk of misunderstandings and loss of nuances compared to using an interpreter. Furthermore, the whole data set was double coded by researchers with different backgrounds. This collaborative coding process helped enhance the credibility of the study’s findings [54].

The participants in this study mainly originated from Syria, had a high education level and were either working or studying. Considering the sociodemographic backgrounds of the participants, the findings in this study may reflect a more liberal view on contraceptives than among Arabic speaking immigrants. While this study provides valuable insights on experiences of contraceptive counselling, we acknowledge that women in more vulnerable situations (for example women with a lower education level, or lower autonomy) might not be represented in this study. However, participants discussed other women’s (friends and families) experiences of contraceptives and contraceptive counselling, not only their own personal ones, which makes the findings more representative for the community.

A challenge faced during the recruitment process was the limited availability of potential participants. It was both difficult to find participants to invite to the study and to get access to some spaces to recruit (for example denied access to recruit at Swedish language school). Additionally, many immigrant women in Sweden might be juggling multiple responsibilities, such as work, education, and family commitments, making it difficult to find the time to participate in the study. To address this issue, the research team offered flexible scheduling options for FGDs, including both in-person and online sessions at different times of the day and in various locations. In retrospect establishing contact with gatekeepers earlier on in the process might have been a successful strategy for recruitment as well as more efforts on snowball sampling. However, the FGDs were heterogenous in terms of country of origin, and the discussions were open, free and displayed a shared understanding, resulting in the data being sufficiently rich to reach saturation [30]. Another challenge was that the participating women largely talked about their experiences of contraceptive counselling in general, rather than only postpartum specific experiences. However, this is expected since postpartum counselling experience is limited as it happens at a certain time and the more general discussion on contraceptive counselling increases the transferability of the findings. Lastly, we did not collect data on when the women gave birth in Sweden. In the initial inclusion criteria, woman should have given birth within the last 2 years in Sweden. However, since it was difficult to recruit, we had to be more flexible. The majority of the women had given birth within in the Swedish health care system recently, but a couple of women had given birth longer ago (5–10 years ago). Consequently, changes in clinical practices may have taken place since their experiences with contraceptive counselling.

## Conclusion

To establish person-centred postpartum contraceptive counselling, health care services need to shift focus from individual barriers to how services are delivered. An open-minded engagement with the patient, and facilitation of informed and free choices are fundamental to support reproductive autonomy. Other strategies might entail introducing the concept of birth spacing- rather than preventing pregnancy- in the postpartum contraceptive counselling. Providing comprehensive verbal and written information in the native language to help women handle conflicting information and ensuring accessible follow-up services are other key aspects. This would make the contraceptive services more equitable.

## Data Availability

No datasets were generated or analysed during the current study.

## Abbreviations

SRHR: Sexual and reproductive health and rights
SRH: Sexual and reproductive health
IUD: Intrauterine device
FGD: Focus group discussion
SDM: Shared decision-making
IMPROVE-it: IMplementing best practice postpartum contraceptive services through a quality imPROVEment initiative for the target group, immigrant women, in Sweden
